# Postural Stability in Athletes during Special Hurdle Tests without a Definite Dominant Leg

**DOI:** 10.3390/ijerph18010172

**Published:** 2020-12-29

**Authors:** Bożena Wojciechowska-Maszkowska, Ryszard Marcinów, Janusz Iskra, Rafał Tataruch

**Affiliations:** Faculty of Physical Education and Physiotherapy, Opole University of Technology, 45-758 Opole, Poland; r.marcinow@po.edu.pl (R.M.); j.iskra@awf.katowice.pl (J.I.); r.tataruch@po.edu.pl (R.T.)

**Keywords:** postural sway, stability, dominant leg, non-dominant leg, hurdles

## Abstract

The purpose of this study was to analyze the body balance control of people walking and hurdling with or without a specific dominant leg in a monopodal position. This study involved 28 physical education students. The center of pressure (COP) was measured with a force plate under four conditions: single-leg standing (right and left) with eyes open and two upper limb positions (the arms were positioned in either a specific hurdle technique manner or alongside the body). A repeated measures analysis of variance (ANOVA) was conducted separately for five parameters of the COP in the medial-lateral (ML) and anterior-posterior (AP) directions under the four conditions. In the single-leg position, athletes without a dominant lower leg had better body balance than those with a dominant lower leg. The position of the upper limbs influenced the body position when hurdling. Accepting the correct position of the upper limbs helped to maintain balance (when overcoming hurdles). In hurdles, the position of the upper limbs should be improved to maintain postural stability and save this function for more demanding postural tasks.

## 1. Introduction

Body balance control is one of the basic elements of sporting techniques and a measure of their effectiveness. Many years of sports training can significantly modify the posture control system and can lead to optimal use of the sensory and motor system modality responsible for body balance of athletes in different sports disciplines [1,2] at various levels of expertise. The results of such research indicate that trained athletes perceive the verticality of the body better than non-trained athletes, and many years of training may weaken the impact of body imbalance stimuli. However, there is much controversy about the static and dynamic balance of athletes. Neuromuscular adaptations achieved through sports training are always specific to the movement being performed [3]. It seems that in the case of athletes, differences in body balance control and postural strategies have the potential to explain the impact of sports training on posture control.

An important issue in the training of sport elites is the repetition of the most effective movement tasks, and the starting point is to control body balance under changing conditions. Body balance control can be assessed using practical or experimental tests in different body positions, e.g., bipedal or single-leg postures [4,5]. Single-leg posture research is mainly focused on determining the differences in body position in the case of dominant and non-dominant legs. Such a situation occurs in hurdling [6,7,8]. In these studies, the dominant and non-dominant legs were determined in different ways, and most often, they were defined with the help of an interview/question and a ball kick with the preferred leg [9,10]. A review of these biomechanical tests shows that the analysis of the technique of arm movements among athletes specializing in hurdling has not yet been conducted. Thus far, the analyses have only concerned lower limbs [7,11].

The experiment presented by Iskra et al. differs from other studies because of the dominant leg identification test [12]. In body balance studies, various tests are often used to determine the dominant leg. These tests are not always consistent with the specifics of a given discipline. Most often, the leg used to perform a kick of a ball is determined as a dominant leg [13,14]. The study conducted by the authors focused on the selection of the group based on a specially prepared test. Iskra et al. conducted research to determine the specific efficiency of the dominant and non-dominant lower limbs and to separate the so-called “two-legged and one-legged” subjects. The results of the relationship between the selection of the attacking limb (leg) in special exercises performed in marching and in motion and the use of this preferred leg during hurdling are described in detail in the paper [12].

The results and successes in hurdling are determined by conditioning (motor) and coordination skills, taking into account, among other things, the ability to maintain rhythm and body balance [15,16]. The body balance when hurdling is important for the efficiency of clearing hurdles, especially for a distance of 400 m. The interdependence of the specific position of limbs (i.e., lower and upper limbs) is an important element of long-term hurdle training and can also form a basis for scientific research [17,18]. The change of the attacker’s leg forms the basis for the development of the so-called “stride pattern”.

The dominance of one of the limbs (left or right) is characteristic of hurdles. Previous observations have been related to competition [17,19,20]. Attempts to link dynamic hurdle exercises (e.g., marching, light jogging, sprinting through the hurdles) with static exercises (e.g., standing or sitting) can justifiably be included in the training methodology [21,22]. Iskra et al. confirmed the relationship between lower limb selection and dynamic exercises of different intensities (running–walking) [12]. Studies looking into the relationship between dynamic and static exercises have not yet been conducted. The choice of the lower limb (“attack leg”) is equivalent to the choice of the upper limb when overtaking a runner (e.g., right leg–left arm). This is the basic principle of hurdles runs and all technical exercises.

### 1.1. Purpose

The aim of this study was to analyze the body balance control of athletes hurdling at a running pace with or without a specific dominant in a monopodal position using two positions of the upper limbs.

### 1.2. Research Questions

(1) What are the differences in the control of body sway for static hurdles between students with and without a specific dominant leg?

(2) Does the position of the upper limbs of students during hurdling impact the control of body balance?

### 1.3. Hypotheses

**Hypotheses** **1.** *The subjects with a dominant limb and without a dominant limb being identified during the hurdles should differ in postural control laboratory tests.*

**Hypotheses** **2.** *It is likely that in a group without an identified dominant leg of the attacker, there will be a more irregular center of pressure (COP) displacement during tests involving both limbs. In a group with a separate attacking limb, differences in postural control in tests performed on the dominant and non-dominant limb will be significant.*

**Hypotheses** **3.** *The position of the upper extremities will affect the body posture among the examined students, and there will be a significant increase in the COP between the upper arms conditions.*

## 2. Materials and Methods

### 2.1. Participants

This study involved 28 men, all physical education students, whose average age was 23.75 years (±1.14 years). The students covered by the research completed 75 h of practical program classes in athletics (3 semesters of studies). The curriculum includes 6 h of practical exercises in hurdling. Data on age and somatic characteristics of the participants are presented in Table 1. The only inclusion criterion was being skilled at hurdling. This ability to hurdle is based on the attitude of the attacker’s dominant (i.e., better) leg in overcoming obstacles [12].

The exclusion criteria for the study subjects were as follows: Lower and upper limb injuries, dizziness, and disease. Before the test, participants were familiarized with the purpose, methodology, and procedures of the test. To take part in the test, they signed a written consent form to participate. The consent was given by the Bioethics Committee of the District Medical Chamber in Opole (Resolution No. 212) and all experiments were conducted in accordance with the Helsinki Declaration.

### 2.2. Functional Test of the Dominant Leg

The criterion adopted was that the taught and measured tasks should be similar. The subjects were of the same age and represented a similar level of training. In this work, we used our own research procedures (i.e., Iskra’s test) [12]. On this basis, two groups were distinguished: single-legged and double-legged. Single-legged included single-leg hurdlers, who crossed the hurdles more often (more than 75% of cases) with the same (better) trail leg (*n* = 12); double-legged included two-legged hurdlers, who crossed the hurdles with both the right and left legs, regardless of the distance between the hurdles and the way they were placed (*n* = 13).

### 2.3. Postural Control Apparatus

Balance measurements were acquired at a sampling rate of 100 Hz in four consecutive 10 s tests with open eyes on a Kistler force plate (Type 9286AA, Kistler Instrente AG, Winterthur, Switzerland). The provisional COP position of the feet was calculated from the recorded substrate reaction forces, which were analyzed in the medial–lateral (ML) and anterior–posterior (AP) directions. On the basis of the COP signal, linear (i.e., range (RA), standard deviation (SD), mean velocity (MV), and frequency (FM)), and nonlinear (i.e., sample entropy (SE)) parameters describing postural balance control were calculated. All tests were performed bare-footed.

The mean of the spectral power frequency (FM) using Welch’s averaged periodogram method (the signal is divided into the longest possible segments to obtain close to, but not exceeding, eight segments with a 50% overlap; each segment is windowed with a Hamming window) was used. The data were not filtered. The signal-to-noise ratio (estimated from the ratio of the standard deviation of COP fluctuation from one participant to the standard deviation of the sample stationary weight placed on the force plate) was more than 10.

### 2.4. Study Procedures for Postural Control Measurements

The students were instructed to stand as motionless as possible; the positions are shown in Photographs 1 and 2. The duration of a single test was 10 s. The COP was measured on a force plate under four conditions: single-leg standing (right and left) with eyes open and single-leg standing with two arm positions (with their arms by their sides and with their arms in a hurdle run position).

**Figure 1:**
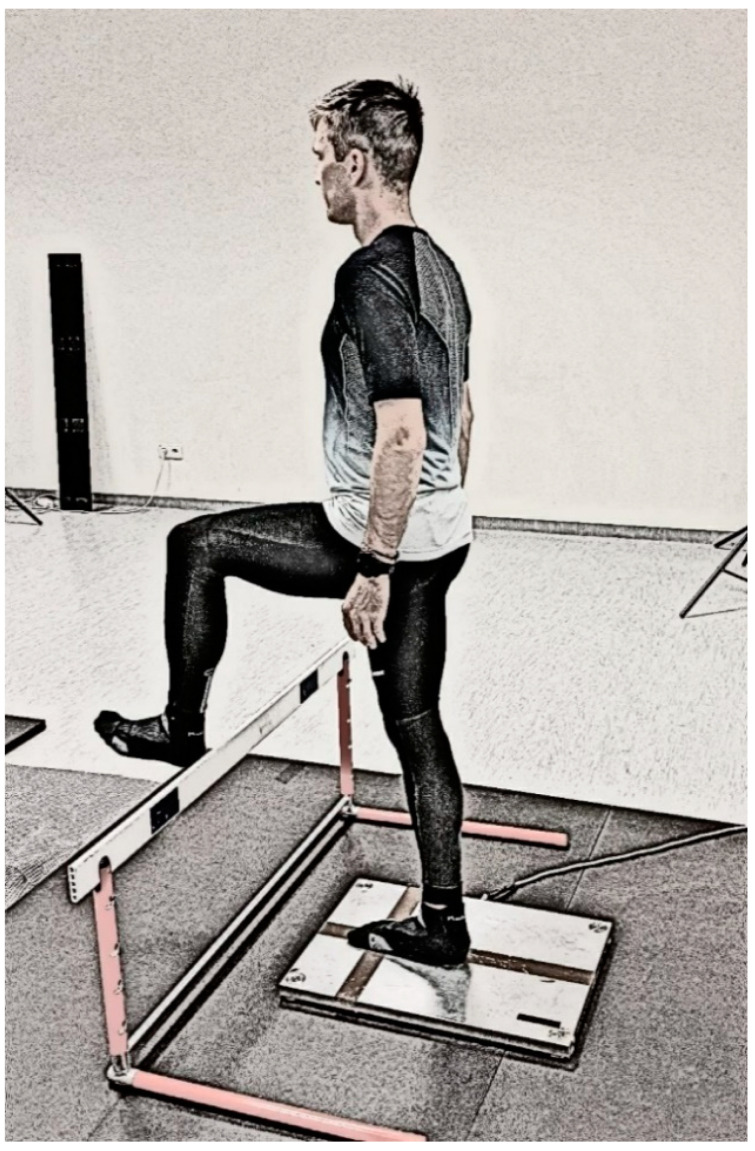
Hurdle attack—arms by the sides.

**Figure 2:**
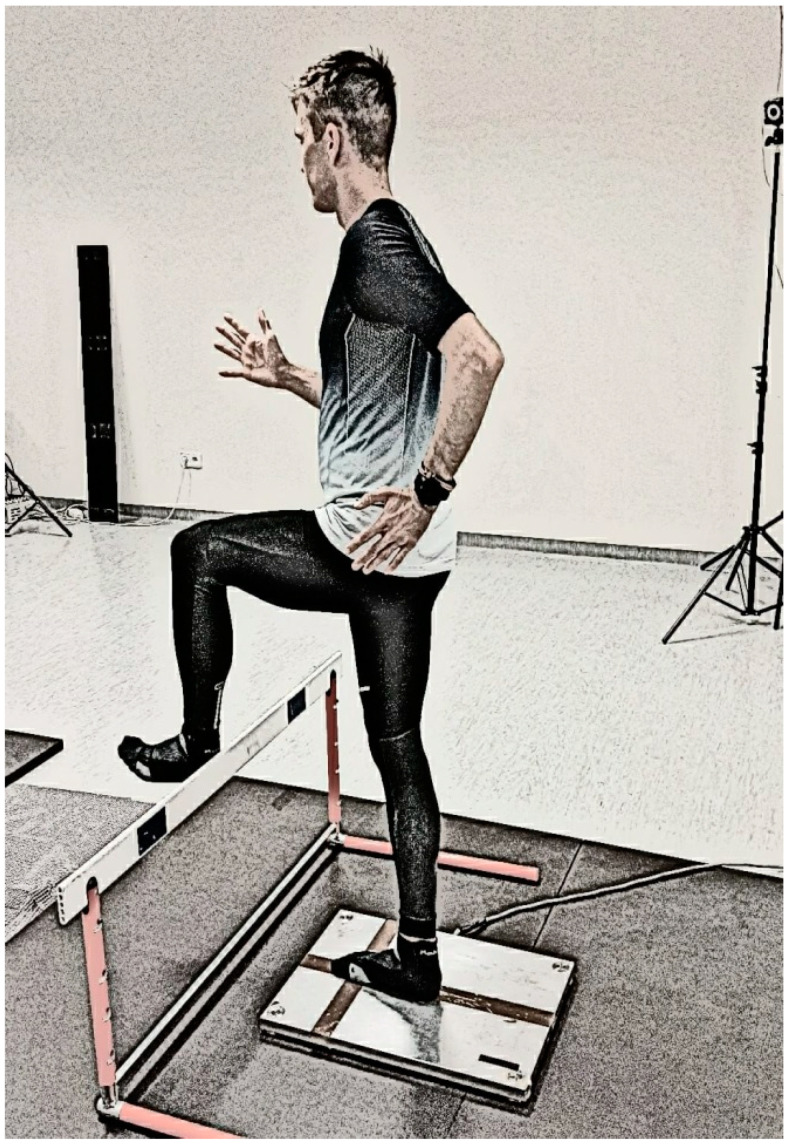
Hurdle attack—arms in classical (hurdle) position.

The hurdle position is a typical opposite position, where an opposite is defeated when the arm of a forward-looking limb is balance the attacking leg (for instance: right attack leg–left arm). H1 refers to a hurdle action without arms (Figure 1), while H2 refers to a hurdle action with arms in the typical position used when clearing hurdles (Figure 2). The position of the hurdle action is similar for both dynamic and static exercises.

For the single-legged group, the single-leg position conditions were as follows: “a”, non-dominant leg—the worst leg in a series of hurdle races; “b”, dominant leg—the best leg (i.e., the attack and take-off leg). In hurdle runs (walks), the athlete has a “dominant” (i.e., better) leg, which attacks successive hurdles.

For the double-legged group, the single-leg position conditions were as follows: “a”, standing on the right lower limb with the left limb up; “b”, standing on the left lower limb with the right limb up.

The measurement started when the participants indicated that they had reached a comfortable and stable position on one leg.

During the test, the thigh of the attacking hurdler was raised to level (parallel to the floor) and the foot was locked into position, directly above the fence. The arm in the “hurdle action” position was directed straight forward, and the other limb was bent at the elbow joint and directed backward.

The tests were performed once in each test position with a 10 s rest between each position, and they were performed in a random order. The test was invalid if the participant moved his standing leg, touched the floor, or used arm movements to regain balance. If the exercise was not performed correctly, the attempt was repeated.

### 2.5. Statistical Methods

All statistical analyses were performed using STATISTICA 13.1 software (StatSoft, Tulsa, OK, USA). Statistical evidence of significance was set at *p* < 0.05. Since the distribution was similar to a normal distribution (Shapiro–Wilk test), analysis of variance (ANOVA) with repeated measures was used to compare the COP parameters across different tasks. The repeated-measure factors included the study design: 2 (experimental group) × 2 (lower limbs (dominant vs. non-dominant or “a” vs. “b”)) × 2 (arm position) × 2 (direction (AP vs. ML)). Post-hoc analyses were performed here with the use of Tukey’s honest significant difference (HSD) test at a statistical significance level of 0.05. Partial eta squared: 0.0099 (small effect size), 0.0588 (medium), and 0.1379 (large).

## 3. Results

**Figure 3:**
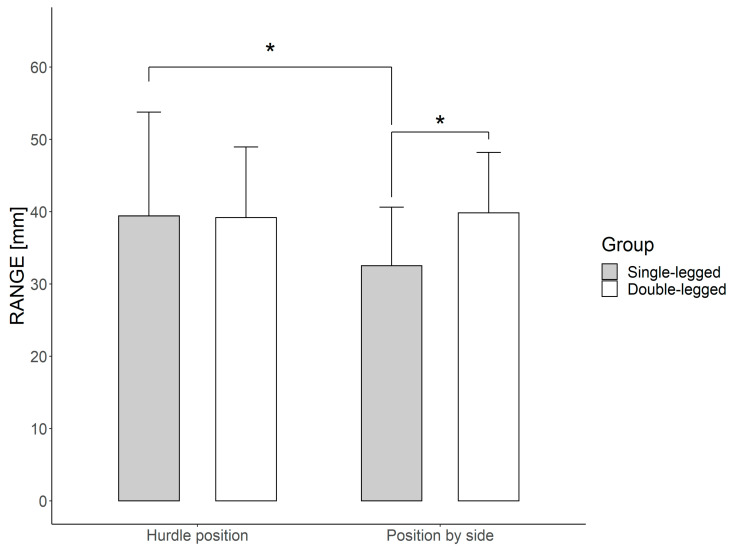
Range (RA) differences (mm) in the anterior–posterior (AP) direction between subjects in the single-legged and double-legged groups (*p* < 0.01) in the one-legged position “b” and under the upper limb condition H2; RA differences (mm) in the AP direction between the upper limb conditions H1 and H2 in group A1 in position “b” (*p* < 0.01). * *p* < 0.01.

Table 2 presents the differences in the COP parameters (i.e., SD, RA, MV, SE, and FM) between groups: single-legged and double-legged. The main effects resulting from the analysis of variance and interactions are presented in Table 3 and Table 4. Post-hoc comparisons are described below and presented in Figure 3.

The ANOVA did not show any significant group effect on the changes in the analyzed COP indicators. The arrangement of upper limbs had a significant impact on the changes in the mean values of COP indicators, i.e., RA, MV, FM, SE. A negligible effect of arm position was found in SD COP. The single-leg standing conditions significantly impacted the changes in SE, whereas the direction of movement had (AP, ML) a significant impact on values RA, SD, FM, and the SE.

The mean velocity showed a relationship between the direction of motion and the fixed position of upper limbs and entropy, between the support leg and the direction of motion, and the direction of motion and the fixed position of upper limbs. The variability of the body amplitude of displacement (SD) is characterized by a relationship between two factors, i.e., the supporting leg and the direction of motion. Tukey’s post-hoc test (HSD) confirmed that the position of the upper limbs had a significant influence on the average body speed of the subjects (single-legged and double-legged groups). A significant increase in MV in the AP direction was observed for the upper limb position H2 in relation to H1 (*p* < 0.0002), while in the ML direction, the same relationship revealed an increase in MV (*p* < 0.0004). Entropy also increased significantly for H2 in relation to H1 in both groups, but only in the AP direction. The post-hoc analysis did not confirm any other dependent influences for group, plane of motion, or standing position on one leg. However, significant interactions were confirmed in the single-legged group. In an attempt to stand in the position in which they attack the hurdle (position “b”), a change in the position of upper limbs from H1 to H2 caused a significant increase in RA in the AP direction (*p* < 0.03). Other differences, confirmed by post-hoc analysis, concerned the students from the single-legged and double-legged groups but were not related to standing position (leg “a” or “b”) or to the upper limb system (H1 or H2). They differed in terms of the RA values between the direction of motion in the AP and ML directions.

## 4. Discussion

The aim of this study was to assess the effect of the upper limb position on the body balance and postural stability in students with and without a dominant leg when hurdling. Body balance control in a single-leg position was measured on the basis of changes in COP indicators. It is assumed that an increase in the value of the linear COP parameters indicates less stability [23,24]. This is true for static balance tests. The range of the COP has been widely used to analyze postural deficits and is a reliable parameter, and larger ranges characterize worse postural stability [5]. A comprehensive review of research results in the area of postural balance revealed that the dominant and non-dominant leg in different groups of athletes (footballers, swimmers, and basketball players) often showed no differences [25].

The measurement time adopted by the authors took into account the specificity of the discipline. The measurement time in the single-leg position adopted by researchers in stabilographic studies varies and is characterized by very short sessions of 5 s [26], with the most commonly used measurement times ranging from 15 s [27,28] to 20–30 s per sample [3,5,13,14,29]. Differences in the measurement times for single-leg standing depend on the purpose of the test, the specificity of the sport, the experience level of the examined competitors, and the difficulty of the task (e.g., its structure). Testing under these conditions is intended to avoid the effect of fatigue if fatigue is not the test target. Studies of changes in the kinematic structure of crossing hurdles under the influence of fatigue require a significant change in equipment and technique [7,30]. The so-called “hurdle step,” i.e., the distance from the rebound before the fence to the landing behind it is then shorter and the time of its execution is longer [16,17].

This study allowed us to determine the nature of changes in body balance control during one-legged hurdles tests. The results presented by the authors show the differences in balance control between the one-legged and two-legged groups and as such they may provide new insights for researchers and trainers. Studies by Prado et al. suggest that people are naturally asymmetrical and exhibit consistent behavior when standing [31]. The issue of interchangeability of the limbs when clearing hurdles is particularly important in 200–400 m hurdles. In a 400 m hurdles race, athletes using one attack leg cover the distance using a “rhythm” (i.e., a stride pattern) with 13.15 (men) or 17 steps (women) [16,20]. Due to increasing fatigue, the running step is shortened, and competitors are forced to increase the number of steps (e.g., from 13 to 14) and thus to change the trail leg, e.g., from right to left [6,19,32,33]. The authors of the training textbooks noted significant differences between the training of athletes with only “one leg” and those who attack hurdles with both their left and right legs [11,15].

Analysis of the mechanisms involved in postural regulation showed lower COP range values in the double-legged than in the single-legged group. It seems that students from the one-legged group in the single-leg position “b” with the upper limb condition H1 have better postural stability in the AP direction than students from the two-legged group. The lower range values in the one-legged group may reflect a lower risk of falling, as the COP is closer to the center of stability. In this group, in the single-leg position of the lower limb system in which they attack the hurdle, a change in the configuration of the upper limbs from H1 to H2 caused a significant increase in the COP range. This confirms the universal principle of teaching running through opposite, which starts the whole process with the right “hurdle position” upper limbs [31]. In the double-leg group, a similar change in the upper limbs did not cause a significant increase in the COP range.

Research by Ibuki et al. showed that a group of dancers had better control in one-legged postures than the control group, i.e., the COP fluctuated more evenly around the COM (Center of Mass) in the AP and ML directions among the dancers than among the controls [29]. However, other reports explain the decrease in the COP range as a greater fear of falling [28,34]. It seems that these explanations are speculative in nature, and further research is necessary, especially in the context of training elite athletes. Postural balance includes task-specific skills that depend on many variables (specifically, on many internal and external variables, i.e., difficulty). The difficulty of the movement when clearing hurdles is evidenced by the significant differences in the body amplitude of displacement (RA) when standing on a support limb (in terms of hurdle back exercises, it is a rebounding limb). From the point of view of training theory, the problem of maintaining balance concerns the sum of motor and coordination dispositions, as well as external conditions [35]. Research-based confirmation of this opinion was obtained in the interactions, taking into account all elements of the statistical analysis (Table 4). The use of the technique of crossing hurdles largely eliminated difficulties in maintaining balance during exercises on the “weaker” lower limb (Figure 3 for the single-legged group). Analysis of the results shows that changes in the leg mainly affected upper limbs. This shows that when clearing hurdles, attention should be paid to the whole system of movement, not only to the work of lower limbs. We observed that the position of upper limbs in athletes clearing hurdles may be crucial in the identification of postural behaviors [12]. Based on a literature review, we know that the influence of upper limb position on body balance in hurdle running has not been addressed by researchers so far. Both scientists and trainers, as well as teachers, point to the relationship between the hurdle clearance technique and upper limb movements [17,18]. Other studies assessing the impact of upper limb positions on body balance control have been included in the discussion. They mainly concern pistol, rifle, and archery athletes [36,37,38,39]. Research conducted on shooters has shown that the rearing of the body affects the effectiveness of shooting with a pistol but not with a rifle. Serrien et al. showed that, for elite archers, it is not necessary to minimize the degrees of freedom of all movements when aiming, but rather to take advantage of the structure of the kinematic chain variation [37]. Mon et al. concluded that balance played different roles in shooting disciplines [39]. Analysis of our data showed that the position of the upper limbs, the so-called “standard” (along the body) most commonly used in stabilographic tests, did not differ in the body balance checks of single- and double-leg competitors. On the other hand, a change in the position of upper limbs according to the specificity of crossing a hurdle affected postural behavior. The position of H2 in relation to H1 significantly increased the average speed in both groups. The significant increase in MV in the AP direction was 5.51 mm/s (from 34.73 to 40.24), while in the ML direction, MV increased by 4.31 mm/s (from 35.37 in H1 to 39.68 in H2). The average speed (MV) reflects the effectiveness of the postural control system (the lower the speed, the better the postural control) and MV is considered to be the most reliable measurement among the studies [5]. High values of MV growth indicate a significant displacement of the point of application of the resulting foot reaction force to the ground. On the basis of the results obtained, it can be confirmed whether the tested subjects are characterized by calm or uncertain control of body balance in the standing position. A more difficult task might force the regulatory mechanism to gather information from the internal senses (e.g., the vestibular or the somatosensory system) that are involved in postural control. Rosker et al. suggested that changing the position of the COM by increasing the difficulty of functional tasks changes the parameters of the COP, including average speed, and can be used in the development of training methods focused on body balance and functionality [40]. Although the experiment did not analyze how and to what extent the COM moves, it can be presumed that changing the position of the upper limbs from H1 to H2 has an impact on the change in the COM and, therefore, modifies postural performance.

Body balance control in stabilographic studies of single-leg standing is qualified for dynamic studies, but not for static studies, as in the case of free standing [5]. The results of the present research also show changes in entropy, which increased significantly in position H2 in relation to H1 in both groups (single-legged and double-legged), but only in the AP direction. Movement in the AP direction is associated with increased activity of the ankle muscles (i.e., the so-called “ankle strategy”) [23]. According to Donker et al. and Roerdink et al., entropy is a measure of irregularity or unpredictability of a time series [9,41]. The authors attribute the decrease in its value to the increased attention paid to the performance of a postural task, while its increase is associated with the automation of a task. According to Paillard et al., a significant regularity in posture control resulting in low entropy (SE) values characterizes systems with reduced adaptability and responsiveness to potential disturbances and an increased risk of falling [5].

### Limitations

Postural behavior, especially in competitive sports, is no longer considered a general ability but rather a specific ability. According to the authors, balance training in highly trained athletes can influence the transfer of motor skills to other related tasks. Due to the complexity of the test procedure, including the definition of the single- and double-leg tests and the conditions of the single-leg test, it seems that the current results cannot be generalized to other tests. Our protocol requires that the subjects have specific motor skills. However, the current data can be used in the study of both less experienced and professional athletes running hurdles and can be used in periodic sports training evaluations.

This is one of the few studies that has examined movements of upper limbs in hurdling. Research has shown that analysis of hurdle runs need to be searched for (e.g., with low manual loading or with analysis before and after run) and test procedures need to be modified (e.g., COM).

Future studies in groups of high-level athletes should focus on identifying differences regarding asymmetry between lower and upper limbs. According to the rules of teaching in complex coordinating competitions and primary sports disciplines (before starting proper dynamic exercises), the task at hand is to establish a static pattern, e.g., the so-called dance “frame,” or, in the case of running hurdles, a proper shoulder position [15,22,42]. For this reason, the help of scientific analyses in the area of static equilibrium is not without significance.

## 5. Conclusions

Students of physical education with athletics specialization using both legs (i.e., the bipedal group) to cross hurdles, with a single-leg position, have better control over their body’s balance than persons crossing hurdles with one dominant lower limb. Additionally, the results of this study indicate that the position of upper limbs in the hurdle posture may result in changes in body deformity among university-level students. In this context, hurdlers must learn to control the balance of their body with different upper limb position configurations according to the requirements of the sport. It is therefore recommended that the training of athletes running hurdles should include exercises for upper limbs to a greater extent. This finding is particularly relevant for people with one dominant leg, in whom changes in the position of the upper limbs during the test caused large changes in balance parameters.

## Figures and Tables

**Table 1:** Characteristics of the subjects (M ± SD).

Participant Characteristics (Groups)		Age (Years)		Height (cm)		Weight (kg)	
	*N*	M	SD	M	SD	M	SD
Single-legged	15	23.80	1.21	176.87	10.90	74.25	11.41
Double-legged	13	23.69	1.11	175.38	8.23	71.85	14.53

Note: M, mean; SD, standard deviation.

**Table 2:** Descriptive statistics for the total sample.

COP Parameters	Direction	Leg	Arms	Groups			
				Single-Legged (*n* = 15)		Double-Legged (*n* = 13)	
				M	SD	M	SD
SD (mm)	ML	A	H1	7.02	2.02	7.30	2.09
			H2	7.11	2.02	7.36	1.73
		B	H1	5.98	1.73	6.91	2.14
			H2	6.80	1.96	7.59	1.25
	AP	A	H1	7.26	1.63	6.90	1.59
			H2	7.12	2.22	7.69	1.93
		B	H1	7.03	1.93	8.40	1.72
			H2	8.07	2.48	7.83	2.01
RA (mm)	ML	A	H1	30.10	6.52	33.41	7.60
			H2	31.82	8.33	33.40	7.05
		B	H1	27.61	7.40	31.26	8.11
			H2	30.79	7.85	34.02	5.95
	AP	A	H1	34.97	8.05	34.12	9.37
			H2	34.73	11.62	40.61	10.69
		B	H1	32.52	8.09	39.83	8.36
			H2	39.39	14.38	39.16	9.80
MV (mm/s)	ML	A	H1	35.20	9.39	36.48	9.99
			H2	41.97	13.45	39.27	9.85
		B	H1	33.87	9.02	35.94	13.72
			H2	37.97	13.35	39.54	8.39
	AP	A	H1	35.55	8.55	34.82	9.50
			H2	43.69	13.38	42.61	12.87
		B	H1	32.47	8.41	36.07	12.60
			H2	40.61	14.04	42.03	12.81
FM (Hz)	ML	A	H1	0.80	0.26	0.79	0.23
			H2	0.93	0.23	0.89	0.33
		B	H1	0.89	0.22	0.83	0.29
			H2	0.88	0.26	0.78	0.20
	AP	A	H1	0.72	0.25	0.71	0.22
			H2	0.84	0.17	0.80	0.28
		B	H1	0.64	0.22	0.67	0.32
			H2	0.67	0.21	0.81	0.27
SE (-)	ML	A	H1	0.75	0.16	0.74	0.16
			H2	0.80	0.14	0.80	0.17
		B	H1	0.79	0.14	0.75	0.19
			H2	0.78	0.14	0.73	0.09
	AP	A	H1	0.99	0.18	1.06	0.23
			H2	1.19	0.16	1.10	0.21
		B	H1	1.01	0.20	0.91	0.27
			H2	1.07	0.28	1.01	0.14

Note: The measures of the center of pressure (COP) are: SD, standard deviation (mm); RA (mm), range; MV (mm/s), mean velocity; FM, frequency; SE, sample entropy; ML, medial-lateral direction; AP, anterior-posterior direction. Groups: single-legged, the single-leg position conditions were as follows: “A” refers to the non-dominant leg, “B” refers to the dominant leg (i.e., the attack and take-off leg); double-legged, the single-leg position conditions were as follows: “A” refers standing on the right lower limb with the left limb up, “B” refers to standing on the left lower limb with the right limb up. H1, with their arms at their sides; H2, with arms in the typical position used when clearing hurdles.

**Table 3:** Main effects of the analysis of variance (η_p_^2^, partial eta squared).

Effect	ANOVA														
	RA (mm)			SD (mm)			MV (mm/s)			SE (-)			FM (Hz)		
	F (1.26)	*p*	η_p_^2^	F (1.26)	*P*	η_p_^2^	F (1.26)	*p*	η_p_^2^	F (1.26)	*p*	η_p_^2^	F (1.26)	*p*	η_p_^2^
Group	1.91	0.18	0.07	0.91	0.35	0.03	0.03	0.86	0.00	0.51	0.48	0.02	0.04	0.84	0.00
Arm	4.79	0.04	0.16	2.09	0.16	0.07	20.84	0.0001	0.44	8.46	0.01	0.25	4.57	0.04	0.15
Leg	0.03	0.87	0.00	0.23	0.64	0.01	1.95	0.17	0.07	4.90	0.04	0.16	1.31	0.26	0.05
Direction	26.67	<0.01	0.51	9.77	<0.01	0.27	1.40	0.25	0.05	193.81	0.00	0.88	16.48	<0.01	0.39

Note: Group: single-legged, double-legged. Arm: with their arms at their sides, with arms in the typical position used when clearing hurdles. Leg: the single-leg position conditions (dominant, non-dominant). Direction: ML, AP.

**Table 4:** Interactions in the analysis of variance (η_p_^2^, partial eta squared).

Interaction	ANOVA														
	RA (mm)			SD (mm)			MV (mm/s)			SE (-)			FM (Hz)		
	F (1.26)	*p*	η_p_^2^	F (1.26)	*P*	η_p_^2^	F (1.26)	*p*	η_p_^2^	F (1.26)	*p*	η_p_^2^	F (1.26)	*p*	η_p_^2^
Arms × Group	0.10	0.75	0.00	0.19	0.67	0.01	0.46	0.50	0.02	0.46	0.51	0.02	0.01	0.94	0.00
Leg × Group	0.24	0.63	0.01	1.39	0.25	0.05	2.23	0.15	0.08	1.78	0.19	0.06	0.11	0.74	0.00
Direction × Group	0.00	0.97	0.00	0.46	0.50	0.02	0.02	0.88	0.00	0.21	0.65	0.01	2.13	0.16	0.08
Arms × Leg	0.17	0.69	0.01	0.24	0.63	0.01	0.19	0.67	0.01	1.43	0.24	0.05	1.41	0.25	0.05
Arms × Leg × Group	1.61	0.22	0.06	1.25	0.27	0.05	0.04	0.85	0.00	0.90	0.35	0.03	0.18	0.68	0.01
Arms × Direction	0.63	0.44	0.02	0.16	0.70	0.01	6.14	0.02	0.19	8.66	0.01	0.25	0.84	0.37	0.03
Arms × Plane × Group	0.05	0.83	0.00	0.16	0.69	0.01	0.14	0.71	0.01	0.75	0.39	0.03	0.42	0.52	0.02
Leg × Direction	2.56	0.12	0.09	5.91	0.02	0.19	0.00	0.97	0.00	4.11	0.05	0.14	1.64	0.21	0.06
Leg × Direction × Group	0.00	0.99	0.00	0.03	0.87	0.00	0.31	0.58	0.01	0.05	0.83	0.00	2.97	0.10	0.10
Arms × Leg × Direction	0.75	0.40	0.03	1.41	0.25	0.05	0.00	0.99	0.00	0.23	0.64	0.01	1.64	0.21	0.06
Arms × Leg × Direction × Group	9.87	<0.01	0.28	3.49	0.07	0.12	2.34	0.14	0.08	2.68	0.11	0.09	0.67	0.42	0.03

Note: Group: single-legged, double-legged. Arm: with their arms at their sides, with arms in the typical position used when clearing hurdles. Leg: the single-leg position conditions (dominant, non-dominant). Direction: ML, AP.

## Data Availability

The datasets generated for this study are available on request to the corresponding author.
